# Value of Blood Cultures in the Management of Children Hospitalized with Community-Acquired Pneumonia

**DOI:** 10.7759/cureus.8222

**Published:** 2020-05-21

**Authors:** Ahmed S Youssef, Mina Fanous, Faisal J Siddiqui, Jorge Estrada, Valeriy Chorny, Melvyn Braiman, Erick F Mayer

**Affiliations:** 1 Pediatrics, State University of New York Downstate Medical Center, New York, USA; 2 Neonatology, State University of New York Downstate Medical Center, New York, USA; 3 Pediatrics, Kings County Hospital, New York, USA; 4 Pediatrics, New York University School of Medicine, New York, USA

**Keywords:** pneumonia, community acquired pneumonia, blood culture

## Abstract

Background and objectives

Current guidelines for the management of community-acquired pneumonia (CAP) in children recommend obtaining a blood culture for children with moderate to severe pneumonia; yet, there is no guidance to assess the severity of the disease. Thus, a blood culture is obtained for the majority of children admitted with CAP, regardless of the severity of their symptoms. The study was designed to investigate and identify the prevalence of bacteremia in pediatric patients hospitalized with CAP and to evaluate the clinical and laboratory variables associated with bacteremia.

Methods

We conducted a medical record review of children aged from two months to 18 years diagnosed with CAP between January 1, 2013, and December 31, 2017, at our two urban tertiary centers. We used binary logistic regression analysis and chi-square tests to look at factors associated with blood culture positivity.

Results

A total of 464 patients were admitted with CAP. Blood cultures were obtained in 357 (76.9%) patients; 23 patients had repeated cultures. Fifteen patients had positive cultures: 5/380 (1.3%) were considered true positive results and 10/380 (2.6%) were considered contaminants. Intensive care unit (ICU) admission (OR 5.6 with 95% CI (1- 31), p<0.03), toxic appearance (OR 12.8 with 95% CI (1.3-125), p<0.01), and significantly elevated C-reactive protein (CRP) (>300 mg/L (p<0.01) were associated with bacteremia.

Conclusion

The prevalence of bacteremia among children admitted for CAP is low. The use of routine blood cultures should be reserved for children with moderate to severe pneumonia. Further studies are required to better risk-stratify children with CAP.

## Introduction

Community-acquired pneumonia (CAP) is one of the main causes of hospitalization of children in the United States, with an annual estimated cost exceeding one billion dollars [1-2]. A recent prospective multicenter study conducted by the Centers for Disease Control and Prevention (CDC) reported that the annual incidence of hospitalization for CAP is 15.7 cases per 10,000 children from January 1, 2010, to June 30, 2012 [3].

Viruses are by far the most common cause of CAP while bacteria account for only 15% of cases [3]. After the introduction of the vaccines against Streptococcus pneumoniae and Haemophilus influenza type B, the rates of CAP secondary to these invasive bacterial infections have decreased from 7.7% to 4% [4-6].

National guidelines for the management of infants and children with CAP, established in 2011 by the Infectious Diseases Society of America (IDSA), recommend "only obtaining blood culture in children with moderate and severe CAP" [7]. This statement, as mentioned in the guideline, fails to accurately define what would be considered "moderate to severe CAP." Past studies have reported a wide range in the prevalence of bacteremia in CAP (0.8%-17.4%) [8-13].

A drawback of routine cultures is a high proportion of blood culture contamination, ranging from 0.7% to 8.1% [14-21]. This results in prolonged hospital stay with subsequent increased cost [18-19]. Blood culture results may influence the management in only 2.2% of the cases [22-24].

A meta-analysis published in 2015 on the role of blood culture in pediatric CAP found that studies focusing on patients with severe CAP had a higher prevalence of bacteremia when compared to studies that included non-severe CAP. Validated clinical prediction rules or stratification criteria to define or categorize the severity of pediatric CAP are not available [14]. In adults, a six-point scoring system based on the presence of confusion, uremia, respiratory rate, blood pressure, and age ≥ 65 years (CURB-65 (confusion, urea, respiratory rate, blood pressure, and 65 years of age or older) score) is widely adopted to define severe pneumonia [25-27]. Few studies have attempted to describe the correlation of clinical or radiological parameters like toxic appearance or presence of effusion with bacteremia secondary to CAP in children [28].

The main study objective was to identify the prevalence of bacteremia in pediatric patients hospitalized with CAP in two tertiary-level hospitals. Secondary objectives included: (1) to determine the prevalence of false-positive blood cultures, (2) to identify the microbiology and susceptibility patterns of true positive blood cultures, (3) to identify the clinical and laboratory variables associated with bacteremia in patients hospitalized with CAP, and (4) to determine the impact of blood culture results on antimicrobial therapy and length of hospital stay.

## Materials and methods

This is a medical record review of all patients admitted to New York City (NYC) Health and Hospitals/Kings County Hospital (KCH) and State University of New York, Downstate Medical Center (DMC) with a diagnosis of community-acquired pneumonia between January 1, 2013, and December 31, 2017. Both centers are tertiary-level hospitals located in Brooklyn, New York. The Institutional Review Board at KCH and DMC approved the study protocol.

We included children and adolescents aged two months to 18 years of age. Subjects were identified according to International Classification of Diseases, Ninth Revision (ICD-9) (480-488.1, 510-511, 513, Jan 1 2013-Sept 30 2015) and ICD-10 (J10-J18.9, Oct 1 2015- Dec 31 2017) codes for pneumonia. We excluded children and adolescents with a diagnosis of hospital-acquired pneumonia, defined as pneumonia diagnosed more than 48 hours from admission or less than two weeks after hospital discharge, as well as children living in chronic care facilities.

Charts were manually reviewed to obtain demographic, clinical, and laboratory data, which included relevant past medical history (previous history of pneumonia, chronic lung disease, asthma, immunodeficiency, sickle cell disease, cerebral palsy), recent use of antibiotics (defined as any patient who self-reported antibiotics use five days before hospitalization ), vaccination status (including influenza vaccine in the last season ), vital signs, general appearance, respiratory examination findings, relevant laboratory results (white blood cell count, neutrophil count, C-reactive protein), imaging studies (chest X-ray, ultrasound, tomography), blood culture (obtained in the first 48 hours upon admission), as well as results of other microbial testing, including nasopharyngeal aspirate and pleural fluid analysis (cell count, pH, protein, glucose, lactate dehydrogenase (LDH), culture). Complications encountered during the hospital course included admission or transfer to the pediatric intensive care unit, supplemental oxygen requirement, and ventilatory support. Length of stay was also recorded.

The distinction between true-positive vs. contaminated blood cultures was determined by the primary team taking care of the patient and later reviewed by two of the authors (AY and EFM).

## Results

We collected data from 464 children. The median age was three years (IQR 2-6) and 51.9% were female. A total of 162 (34.9%) had no significant past medical history and 446 (96%) were up-to-date on their immunizations, as shown in Table 1. Table 1 outlines the demographic and clinical characteristics of our population.

**Table 1:** Demographic and clinical characteristics of children admitted with CAP CAP: community-acquired pneumonia; SMA-1: spinal muscular atrophy; PMH: past medical history; PCV: Pneumococcal vaccine; HiB: Haemophilus influenzae type b; ER: emergency room; NIV: noninvasive ventilation

Demographic and Clinical Characteristics	
Gender	Male: 222
	Female: 241
Medical history	Asthma: 209
	Sickle cell disease: 40
	Cerebral palsy: 15
	Immunodeficiency: 3
	Seizures disorder: 6
	Prematurity: 6
	SMA-1: 2
	Others: 16
	No significant PMH: 162
Vaccination	PCV: 446
	HiB: 446
	Influenza: 285
Received antibiotics prior to admission	Total: 64
	Amoxicillin: 41
	Macrolides: 11
	Cephalosporins: 7
	Penicillin: 4
	Bacterium: 1
Clinical examination	Fever: 270
	Crackles: 142
	Wheezes: 175
	Retractions: 208
	Decreased air entry: 140
PICU admission	Directly admitted from ER: 108
	Transferred from the regular ward: 16
Respiratory support	Oxygen by nasal cannula: 134
	NIV 2: 60
	Intubation: 8
Radiographic findings	Focal consolidation: 311
	Perihilar opacity: 76
	Atelectasis effusion /necrotizing: 18
	Normal: 19
	No chest X-ray: 40
WBC 10^3^/µL; CRP mg/L	< 15 000: 361
	>15 000: 103
	< 5: 16
	5-10: 6
	10-50: 33
	50-100: 18
	100-200: 24
	200-300: 9
	>300: 3
First-line Antibiotic	Ampicillin: 178
	Cephalosporins: 199
	No antibiotics: 22

A total of 380 blood cultures were obtained, including 23 patients who had repeated blood cultures. Fifteen (3.9%) cultures were positive for bacterial growth; five (1.3%) were considered true positives while the other 10 (2.6%) were treated as contaminants. The prevalence of bacteremia was 1.3% in those with blood cultures and 1.0% in all patients admitted with CAP (Table 2). Except for one patient who had human immunodeficiency virus (HIV) with low cluster of differentiation 4 (CD4) count, none of our bacteremic patients had immunodeficiency, indwelling central line, or sickle cell disease; however, two of them had a history of asthma and prematurity. Out of the five cases of bacteremia, three were caused by S. pneumoniae, one caused by methicillin-resistant, Staphylococcus aureus, and one by Enterococcus avium.

**Table 2:** Demographic, clinical, laboratory, and radiological characteristics of patients with bacteremia HIV: human immunodeficiency syndrome; AIDS: acquired immunodeficiency syndrome; CD4: cluster of differentiation 4; HAART: highly active antiretroviral therapy; MRSA: methicillin-resistant Staphylococcus aureus; ARDS: acute respiratory distress syndrome; NICU: neonatal intensive care unit; CLD: chronic liver disease; CPAP: continuous positive airway pressure; BIPAP: bilevel positive airway pressure

No	Age	PMH	Cultures	Hospital course	Toxic	ICU	WBC 10^3^/uL	CRP mg/L	CXR	Antibiotics
1	18 years	Congenital HIV AIDS (viral load 6,270,000, CD4 2.59, noncompliant with HAART 1 and azithromycin)	Blood culture: MRSA. Tracheal aspirate Culture: MRSA. Sputum Culture: candida albicans	ARDS+septic shock 2	Yes	Direct admission	3.24	N/A	Cavitary lesion	Initial:-vancomycin, piperacillin/ tazobactam, and azithromycin. Final: -the patient was transferred to another hospital
2	5 months	Preterm 34 weeks NICU for 1 month. No Hx of CLD 3.	Blood culture: Enterococcus avium sensitive to Ampicillin.	Needed CPAP 4, positive blood culture treated as true infection	Yes	Direct admission	10.2	104	Mild pleural effusion. Did not need tapping	Initial­:- vancomycin, ceftriaxone, azithromycin. Final: - ampicillin.
3	6 years	Moderate persistent asthma	Blood culture: Streptococcus pneumoniae	Improvement In the first 24 hours. -But delay in discharge waiting for second culture results	No	No	15.3	N/A	Focal consolidation	Initial: - ampicillin. Final: - ampicillin
4	8 years	None	Blood culture: Streptococcus pneumoniae	Improvement in the first 24 hours. But unnecessary antibiotic upgrade and delay in discharge	No	No	30	N/A	Focal consolidation	Initial: - ampicillin and azithromycin; later changed to ceftriaxone based on culture results. Final: - amoxicillin
5	3 years	None	Blood culture: Streptococcus Pneumoniae. Pleural fluid culture: no growth.	Needed BIPAP 5, and right-side decortication	No	Transferred on the second day of admission	17	321	Large pleural effusion	Initial ampicillin; later changed to ceftriaxone and vancomycin. Final: - amoxicillin

Three out of five bacteremic patients were admitted to the pediatric intensive care unit (PICU). Two were admitted directly from the emergency department (ED) to the PICU and were treated aggressively with broad-spectrum antibiotics. One patient was initially treated with ampicillin in the inpatient unit but deteriorated clinically and was transferred to the PICU, where antibiotic treatment was advanced to ceftriaxone and vancomycin.

Two patients who were admitted to the inpatient unit were started on ampicillin and continued to show improvement on the same antibiotic. We found a significant association between PICU admission (OR 5.6 and 95% CI (1.0-31), p<0.03) and ill appearance on presentation (OR 12.8 and 95% CI (1.3-125), p<0.01) with a true-positive blood culture.

Ten out of 15 positive cultures (67 %) were contaminated: three of the contaminant cultures grew Streptococcus viridans, two grew Staphylococcus hominis, one culture grew both Acinetobacter baumannii and Corynebacterium striatum while the remaining grew Staphylococcus haemolyticus, Staphylococcus simulans, and Rothia spp. Fifty percent of the false-positive cultures were repeated. (Table 3).

**Table 3:** Demographic, clinical, laboratory, and radiological characteristics of patients with false-positive (contaminated) blood cultures WBC: white blood cell; CXR: chest X-ray; CRP: C-reactive protein; ED: emergency department

No.	Age	Vaccination	PMH	ICU	WBC 10^3^/^27^ul	CXR	CRP mg/L	Hospital course	Cultures	Antibiotics
1	20 months	Yes	None	No	22.5	N	No	Hospital stay: 21 hours.	Blood culture: Staph simulans	Initial: Amoxicillin for 1 day. Final: Amoxicillin 10 days as an outpatient
2	19 months	No	None	No	11.8	N	No	Hospital stay 50 hours	Blood culture: Staph Hominis	Initial: Ceftriaxone for 1 day. Final: Amoxicillin for 10 days as an outpatient
3	3 years	Yes	None	No	8.7	N	No	Hospital stay 24 hours	Blood culture: Strep Viridans	Ampicillin one dose in the ED
4	6 years	Yes	Mild persistent asthma	No	17.1	N	No	Hospital stay 1 day and 21 hours	Blood culture: Staph Hominis	Initial: Ampicillin one dose in the ED. Final: Amoxicillin for 10 days as an outpatient
5	4 months	No	None	No	27.1	37.3	No	Hospital stay: 2 days,15 hours Inpatient team impression that blood culture is contaminant	Blood culture: Strep Viridans Urine culture: Pseudomonas.	Initial: ceftriaxone for 2 days. Final: cephalexin for 10 Days as an outpatient.
6	4 years	Yes	None	No	9.9	N	No	Hospital stay 1 day, 5 hours	Blood culture: Rothia Dentocariosa	Ceftriaxone one dose in the ED
7	2 years	Yes	None	Yes	7	N	No	PICU for 13 hours, inpatient team impression it is a contaminant, but continued antibiotics. Normal Echo. Total hospital stay: 3 days,13 hours.	Blood culture: Strep viridans	Initial: Ceftriaxone for 1 day then ampicillin for 2 days. Final: amoxicillin for 7 as an outpatient
8	2 years	No	Williams syndrome. Mild persistent asthma. Has tracheostomy	Yes	15	7.7	No	treated for the Serratia marcescens infection. Blood culture considered a contaminant. Total hospital stay: 4 days,21 hours	Blood culture: Acinetobacter baumanni+ Corynebacterium Striatum. Tracheal aspirate culture: Serratia marcescens RVP1: RSV	Ceftriaxone for 3 days. Then cefixime for 2 days
9	16 years	No	Cerebral palsy G-tube. Scoliosis	Yes	11.2	N	No	Treated as Aspiration pneumonia. 1 day in PICU, NIV not needed. Total hospital stay: 4 days, 1 hour.	Blood culture staph capitis	Ceftriaxone + Ampicillin/Sulbactam for 1 day. Then Amoxicillin+ clavulanic acid for 4 days
10	7 years	Yes	Intermittent asthma	No	5.7	N	No	Hospital stay: 7 hours.	Blood culture: Staph haemolyticus	Ampicillin one dose in the ED

Finally, in regard to the radiological and laboratory investigations. Of 19 (4%) patients with evidence of effusion or necrotizing pneumonia on chest radiographs, three out of the 19 patients had positive blood cultures; and out of these three, only one patient underwent pleural fluid tapping. Two additional patients who underwent pleural tapping had negative blood cultures, and none of these three patients who undergone tapping had positive pleural fluid cultures. The radiographic evidence of effusion or necrotizing pneumonia was associated with bacteremia (P<0.11) while the binary logistics regression analysis showed that significantly elevated CRP (> 300 mg/L) was associated with a true-positive blood culture (p<0.01).

## Discussion

Bacteremia is an unusual complication of CAP in hospitalized children. Our study suggests that approximately 1% of admitted children with CAP are bacteremic. Penicillin-susceptible S. pneumoniae was the most common isolated organism (60%), which is consistent with recently published studies [15]. Patients with bacteremia mostly had co-morbid conditions, were ill-appearing, or were admitted to the PICU. Our findings are consistent with current IDSA guidelines, which suggest that only children with moderate to severe CAP will benefit from obtaining a blood culture on admission.

Unlike some reports, we did not find a correlation between the radiological findings of effusion or necrotizing pneumonia and a truly positive blood culture. Heine et al. found that five out of 155 children who were admitted with pneumonia to the Children's Hospital at the Medical University of South Carolina or discharged from the ED were bacteremic; all five cases had parapneumonic effusions [28]. Heine et al. found that five out of 155 children who were admitted to the Children's Hospital at the Medical University of South Carolina with pneumonia were bacteremic and all of them had parapneumonic effusions. Similarly, Kwon et al. found a low prevalence of bacteremia in 2705 previously healthy children and adolescents. Of 2705, only three children (0.11%) had true-positive results, and all of them had pneumonia complicated with pleural effusion [29]. Another study by Myers et al. reported that children who required pleural drainage procedure or had a distant site infection had higher rates of bacteremia - 21% and 75%, respectively [11].

Toxic appearance and admission or transfer to the PICU were associated with bacteremia. This is consistent with prior reports from Heine et al., where four out of five children with CAP complicated by bacteremia required ICU care.

The impact in the management of the blood culture results was limited in our study. Two patients were admitted directly to the ICU while a third one was transferred from the inpatient unit after clinical deterioration. The results from the blood culture allowed for accurate diagnosis in all of them and appropriate antibiotic de-escalation at least in two of the patients.

For the two other patients admitted to the inpatient unit, these results led to a therapeutic dilemma with subsequent prolongation of hospital stay for both of them and unnecessary broadening of antibiotics for one of them, even though the reported organism was penicillin-susceptible S. pneumoniae.

The prevalence of blood culture contaminants in this study was 2.6%, which is similar to prior reports [28]. Furthermore, a positive blood culture was twice more likely to be a contaminant than a true pathogen. Despite the high likelihood of reported growth in blood culture to be a contaminant, determining the possibility of true bacteremia is still challenging for physicians and impacts negatively on patient care. In this study, five of the 10 patients with false-positive cultures had repeated cultures, even though the treating physician's impression was in favor of a contaminant organism.

This study has several limitations. Our research is a retrospective chart review that is more prone to chart abstraction biases, especially personal data such as ill appearance. This study was done in our two urban academic medical centers and its results may not be generalizable to other institutions. Finally, we do not have data on whether children discharged from our two facilities may have been readmitted to other hospitals.

## Conclusions

The rate of positive blood cultures in children admitted with CAP is low, and its impact on clinical management is limited. Children that were ill-appearing, admitted to the intensive care unit, or with significantly elevated CRP levels were more likely to have bacteremia secondary to CAP. The current recommendation of obtaining a blood culture for children with moderate to severe CAP, although correct, needs to be clarified. Further studies are needed to better risk-stratify children with CAP, thus providing a targeted approach for obtaining blood cultures, standardizing management, and potentially reducing the cost and length of hospital stay.
